# Mec1 Modulates Interhomolog Crossover and Interplays with Tel1 at Post Double-Strand Break Stages

**DOI:** 10.4014/jmb.1909.09020

**Published:** 2019-12-04

**Authors:** Min-Su Lee, Jung Whan Joo, Hyungseok Choi, Hyun Ah Kang, Keunpil Kim

**Affiliations:** Department of Life Sciences, Chung-Ang University, Seoul 06974, Republic of Korea

**Keywords:** Mec1, Tel1, meiosis, recombination, double-strand break

## Abstract

During meiosis I, programmed DNA double-strand breaks (DSBs) occur to promote chromosome pairing and recombination between homologs. In *Saccharomyces cerevisiae*, Mec1 and Tel1, the orthologs of human ATR and ATM, respectively, regulate events upstream of the cell cycle checkpoint to initiate DNA repair. Tel1^ATM^ and Mec1^ATR^ are required for phosphorylating various meiotic proteins during recombination. This study aimed to investigate the role of Tel1^ATM^ and Mec1^ATR^ in meiotic prophase via physical analysis of recombination. Tel1^ATM^ cooperated with Mec1^ATR^ to mediate DSB-to-single end invasion transition, but negatively regulated DSB formation. Furthermore, Mec1^ATR^ was required for the formation of interhomolog joint molecules from early prophase, thus establishing a recombination partner choice. Moreover, Mec1^ATR^ specifically promoted crossover-fated DSB repair. Together, these results suggest that Tel1^ATM^ and Mec1^ATR^ function redundantly or independently in all post-DSB stages.

## Introduction

During meiosis, duplicated chromosomes undergo recombination, wherein they exchange genetic material and form recombinants called crossovers (COs), which are essential for genetic diversity. In the first meiotic division, homologous recombination yields COs that are also required for appropriate chromosome segregation [1]. Pre-meiotic DNA replication occurs—before initiation of meiosis—yielding sister chromatids tightly held by the cohesin complex to prevent premature chromosome segregation. Parental chromosomes are aligned to form the synaptonemal complex (SC), a meiosis-specific chromosome structure playing essential roles in accurate chromosome segregation and meiotic recombination. Recombination occurs at the central regions of the SC via an association between the homologous chromosomal axis (maternal and paternal) and maturation/formation of the chromosome axis [2-4]. Meiotic recombination is initiated by programmed double-strand breaks (DSBs) generated by the type II topoisomerase-like transesterase, Spo11 [5-7], which interacts with diverse accessory factors (Rec102, Rec104, Rec114, Mei4, Ski8, and Mer2) to regulate the cellular machinery for DSB formation [8]. Thereafter, DSBs are processed by the endonucleolytic protein complex Mre11-Rad50-Xrs2 [9, 10], and the 5’ ends of DSBs are further resected by Exo1, Dna2, and the Sgs1-Top3-Rmi1 complex to expose 3’ single-stranded DNA tails [9], which are coated by replication protein A (RPA) complex to prevent the formation of DNA secondary structures and form nucleofilaments with RecA homologs Dmc1 and Rad51 [11-13]. These nucleofilaments containing RecA homolog-ssDNA complexes invade templates, and are stabilized in single-end invasions (SEIs) and double Holliday junctions (dHJs) and are finally processed to form crossovers (COs) [2, 13 ,14]. Other species are processed as interhomolog non-crossovers (NCOs) without the exchange of flanking genes [14].

Mec1 and Tel1 in budding yeast, also known as ATR and ATM in mammals, respectively, are involved in the meiotic checkpoint network, DSB formation, and recombination [15-17]. Tel1^ATM^ and Mec1^ATR^ are serine/threonine kinases that induce phosphorylation at Ser or Thr residues preceding Gln residues, which are called SQ/TQ motifs. Thus, many Mec1^ATR^ and Tel1^ATM^ targets include SQ/TQ cluster domains (SCDs) [18]. During meiosis, Mec1^ATR^ and Tel1^ATM^ phosphorylate Hop1-T318 in Hop1 SCD and affect the activity of Hop1, a regulator of meiotic interhomolog recombination [17, 19]. Moreover, in budding yeast, Mec1^ATR^ localizes to replication protein A (RPA)-coated ssDNA sites via Ddc2 [20]. Tel1^ATM^ is activated upon DSB formation and is recruited to unresected DSBs by the MRX complex [21-23]. Moreover, phosphorylation of adaptor-proteins by Tel1^ATM^ and Mec1^ATR^ activates downstream effector kinases during meiosis [24]. In this study, we analyzed the roles of Tel1^ATM^ and Mec1^ATR^ in meiotic recombination via DNA physical analysis.

## Materials and Methods

### Yeast Strains

**Fig. 1:**
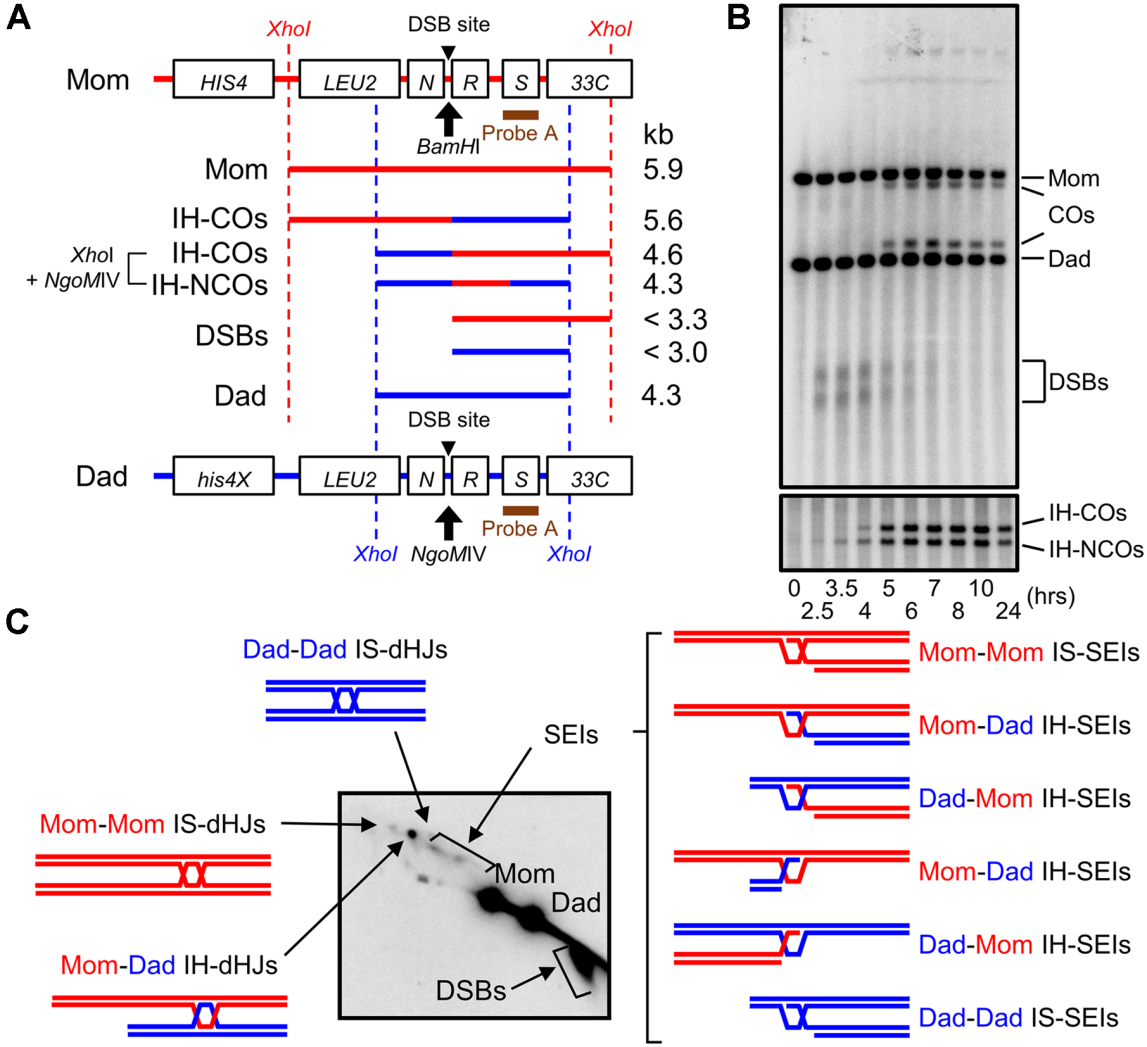
Physical assay for meiotic recombination. (**A**) Physical map of the *HIS4LEU2* locus [2, 43]. Probe A was used to detect DNA fragments via Southern blot analysis. DSB, double-strand break; CO, crossover; NCO, noncrossover. (**B**) Representative image of 1D gel analysis and CO/NCO analysis. (**C**) Representative image of twodimensional gel analysis. dHJs, double-Holliday junctions; SEIs, single-end invasions.

Yeast strains used herein were derived from SK1. They were homozygous for *MATa/MATα* (Table S1). The *HIS4LEU2* locus and restriction sites are shown in Fig. 1.

### Meiotic Time Course

Diploid cells were plated on a YPG plate (1% yeast extract, 2%peptone, 3% glycerol, and 2% bacto-agar) and incubated at 30°C. Cells were streaked on a YPD plate (1% yeast extract, 2% peptone, 2% glucose, and 2% bacto-agar) and incubated at 30°C. A single colony was inoculated in 2 ml YPD liquid medium (1% yeast extract, 2% peptone, and 2% glucose) and cultured for 24 h at 30°C [25, 26]. To synchronize cells in the G1 stage, cultured cells were diluted 1:500 with SPS medium (1% potassium acetate, 1%peptone, 0.5% yeast extract, 0.17% yeast nitrogen base without amino acids, 0.5% ammonium sulfate, and 0.05 M potassium biphthalate; pH 5.5) and cultured for 18 h at 30°C in a shaking incubator. Synchronized cells were washed with SPM medium (1% potassium acetate, 0.02% raffinose, and 150 µl/l antifoam) at the same volume. To initiate meiosis, synchronized cells were resuspended in SPM medium and harvested at each time point and cross-linked with 0.1 mg/ml trioxalen via UV irradiation at 365 nm for 15 min.

### DNA Physical Analysis and Southern Blotting

Genomic DNA was extracted and physical analysis of DNA was performed as reported previously [2, 12]. For one-dimensional (1D) analysis, DNA was digested with XhoI for 3 h at 37°C. Digested DNA fragments were loaded on a 0.6% Seakem LE agarose gel—without ethidium bromide (EtBr)—prepared in TBE buffer (89 mM Tris, and 2 mM EDTA, pH 8.3), and run at ~2 V/cm for 24 h. For CO/NCO gel electrophoresis, genomic DNA was digested with XhoI and *NgoM*IV at 37°C. Digested DNA fragments were loaded onto a 0.6% agarose gel. For twodimensional (2D) analysis, digested DNA fragments were loaded onto a 0.4% gold agarose gel without EtBr and run at ~1 V/cm for 21 h. The gel was stained with EtBr (0.5 μg/ml) for 30 min, and the lanes of interest were excised and placed in a 2D gel tray. 2D gel separation was carried out using 0.8% agarose gel containing EtBr (0.5 μg/ml) (prepared in TBE) at ~6 V/cm for 6 h at 4°C. Thereafter, the gel was treated with 0.25 M HCl for 20 min and with 0.4 M NaOH for 30 min, and then transferred to Zeta-probe membrane (Bio-Rad). Southern blot analysis was carried out using ^32^P-dCTP-labeled radioactive nucleotides treated with a Random Primer Labeling Kit (Agilent Technologies). Signals corresponding to hybridized DNA fragments were detected using PhosphoImager (Bio-Rad) and quantified using Quantity One (Bio-Rad).

### Assessment of Meiotic Division

Meiotic cells from SPM cultures were harvested and stored in 0.1 M sorbitol containing 40% ethanol at −20°C. To visualize and count cells in meiosis I and II, cells were stained with 4’,6- diamidino-2-phenylindole (DAPI) and visualized using an Olympus BX53 fluorescence microscope (Olympus, Japan).

## Results and Discussion

### DNA Physical Analysis for Meiotic Recombination

Meiotic recombination intermediates were analyzed using one-dimensional (1D) and two-dimensional (2D) gel electrophoresis of the *HIS4LEU2* locus, which is a well-characterized hot spot (Fig. 1) [2, 27-29]. Cells synchronized in the G1 phase were harvested at indicated time points. To crosslink DNA inter-strands, cells were treated with Psoralen and exposed to UV irradiation. Genomic DNA was extracted and digested with 60 units of XhoI enzyme. Digested genomic DNA fragments were separated via 1D and 2D gel analysis, followed by Southern blot hybridization (Fig. 1). DSBs were detected at ~3.0 and ~3.3 kb via 1D gel electrophoresis (Figs. 1A and 1B). Joint molecules (JMs) were detected using native-native 2D gel analysis and distinguished on the basis of their conformation and molecular weight (Fig. 1C) [2, 13, 27]. CO and NCO products at the *HIS4LEU2* locus were digested by XhoI and *NgoM*IV. CO and NCO were detected at 4.6 kb and 4.3 kb via 1D gel electrophoresis (Figs. 1A and 1B) [2, 14].

### Tel1^ATM^ Restricts DSB Levels

**Fig. 2:**
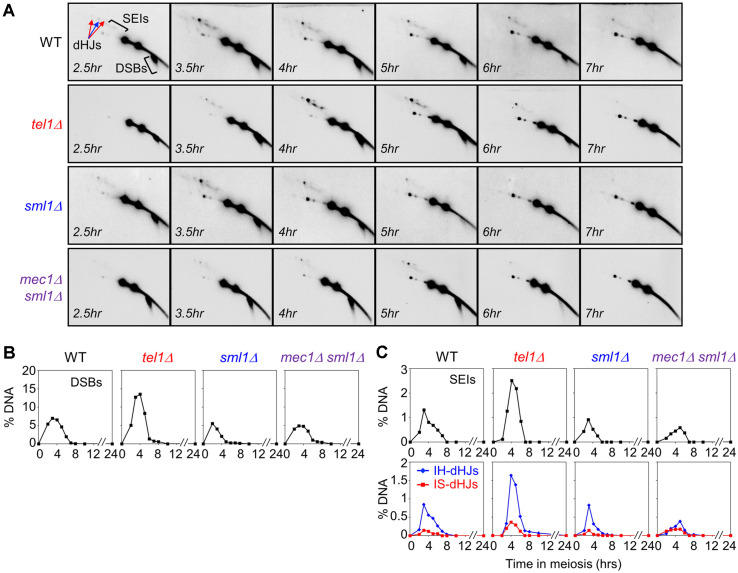
Analysis of recombination intermediates in WT, *tel1Δ*, *sml1Δ*, and *mec1Δ* *sml1Δ* cells. (**A**) 2D gel analysis at each time point in WT, *tel1Δ*, *sml1Δ*, and *mec1Δ* *sml1Δ* double mutant cells. (**B**) Quantitative analysis of DSBs during meiotic recombination. (**C**) Quantitative analysis of SEIs and dHJs in WT, *tel1Δ*, *sml1Δ*, and *mec1Δ* *sml1Δ* double mutant cells.

Meiotic DSBs are induced by Spo11 and its accessory factors, and repaired via recombination [5, 17]. Tel1^ATM^ suppresses clustered meiotic DSB formation and Spo11 via a negative feedback mechanism [30, 31]. Further, it has been reported that chromosome-based Tel1^ATM^ negatively regulates DSB formation by *trans* inhibition activity [30]. Thus, to investigate the roles of Mec1^ATR^ and Tel1^ATM^ in DSB formation, we assessed the recombination process via DNA physical analysis at the *HIS4LEU2* hotspot on chromosome III in the absence of Mec1^ATR^ and Tel1^ATM^. The absence of Mec1^ATR^ causes cell death via cell cycle arrest, suppression of DNA replication, and response to DNA damage [33, 34]. To overcome cell growth defects in the absence of Mec1^ATR^, we introduced a deletion in *SML1*, which decreased the resistance to DNA-damaging agents and DNA replication defects by suppressing dNTP synthesis and inhibiting interactions with ribonucleotide reductase, Rnr1, to examine the function of Mec1^ATR^ [35]. In WT cells, DSBs were initiated at 2.5 h and peaked (~7.5%) at 3.5 h before gradually reducing in number. In *sml1Δ* and *sml1Δ mec1Δ*, DSBs were initiated at 2.5 h and peaked (~7%), being slightly reduced in comparison with that in WT cells; however, these mutants displayed similar DSB levels. In the absence of Tel1^ATM^, DSBs appeared at 2.5 h, in a manner similar to that in WT cells, while the number of DSB at 4 h increased to 2-fold (~14%) in *tel1Δ* cells in comparison with that in WT cells (Figs. 2A and 2B). Through DNA physical analysis, we observed that levels of DSB at the *HIS4LEU2* locus were markedly increased in the absence of Tel1^ATM^. Spo11 and its accessory factors were regulated by Mec1^ATR^ and Tel1^ATM^, which are required for the DNA damage response with the MRX complex [36], which in turn is involved in meiotic recombination and required for Tel1^ATM^ localization at the DSB sites [21,37-39]. Thus, high DSB levels in *tel1Δ* cells may have resulted from unregulated Spo11 and MRX along with other accessory factors modulating the Spo11-oligonucletide complex at early stages of meiotic recombination.

### Absence of Mec1^ATR^ Reduces Levels of IH-JMs

Homologs rather than sister chromatids are preferred as templates at DSBs during meiotic recombination [2]. In contrast, the biological importance of mitotic DSB repair is inter-sister recombination. To assess the formation of JMs in the absence of Mec1^ATR^ and Tel1^ATM^ during meiotic recombination, we performed 2D gel analysis in each single and double mutant. In WT cells, SEIs reached 1.5% and the IH:IS-dHJs ratio was 5:1. In *tel1Δ* cells, the levels of JMs including IH-dHJs and IS-dHJs were significantly increased in comparison with those in WT, and the ratio of IH:IS-dHJs was ~5:1 (Fig. 2). In the absence of Sml1, DSBs, SEIs, and dHJs exhibited a pattern similar to that observed in WT cells; however, in the *sml1Δ mec1Δ* strain, the level of IH-dHJs was reduced, while that of IS-dHJs was comparably increased. Moreover, the IH:IS-dHJ ratio was altered to ~2:1 in the absence of Sml1 and Mec1^ATR^ (Figs. 2A and 2C; Fig. S1). Thus, Mec1^ATR^ promotes interhomolog interaction to form IH-dHJ, while Tel1^ATM^ regulates DSB formation. During meiosis, the biological imperative is inter-homolog recombination, although sister chromatids are present. To promote this phenomenon, Mec1^ATR^ and its interacting proteins potentially regulate the molecular system to govern IH recombination. Hop1, an axial element protein, is a downstream target of Mec1^ATR^. Phosphorylated Hop1 promotes chromosomal localization and activation of Mek1, which also promotes IH interaction in meiotic recombination. Therefore, Mec1-mediated Hop1 phosphory-lation might promote IH formation by Rad51 activity in meiotic mode, which is regulated by Mek1 phosphorylation and recruitment.

### Mec1^ATR^ Promotes CO-Designated Recombination

**Fig. 3:**
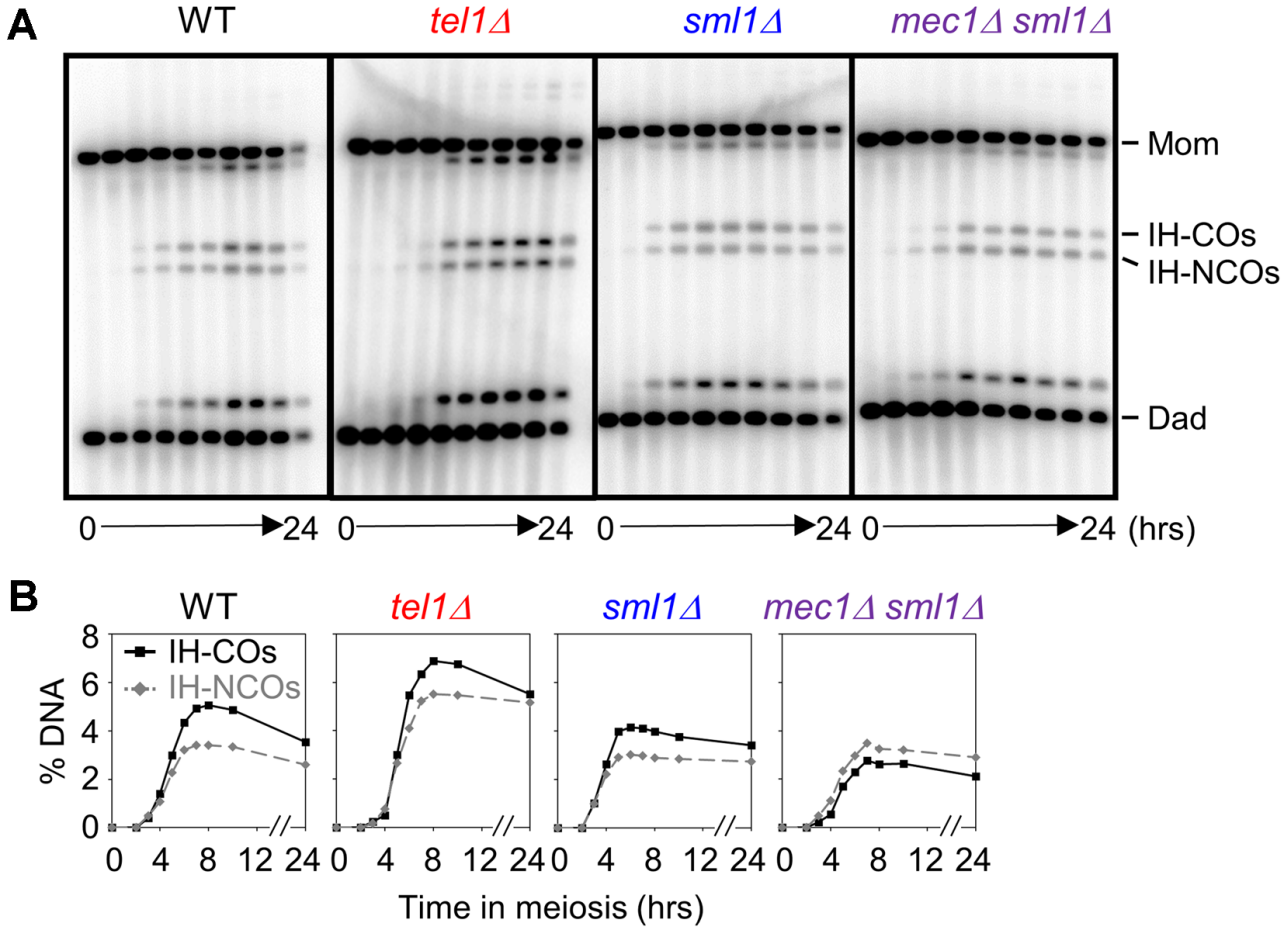
Analysis of CO and NCO in WT, *tel1Δ*, *sml1Δ*, and *mec1Δ* *sml1Δ* cells. (**A**) Representative CO/NCO gel images for WT, *tel1Δ*, *sml1Δ*, and *mec1Δ* *sml1Δ* double mutant cells. (**B**) Quantitative analysis for CO and NCO.

The formation of COs requires SC formation, which stabilizes the progression of IH-SEIs to promote CO-designated DSB repair. Mec1^ATR^ and Tel1^ATM^ phosphorylate Hop1, thus stabilizing the SC via formation of the chromosome axis [17]. To investigate whether Mec1^ATR^ and Tel1^ATM^ are involved in both CO and NCO recombination, we examined physical analysis of recombination in mutant strains. In WT cells, the maximum levels of CO and NCO species were ~5% and ~3.5%, respectively. In the absence of Tel1^ATM^, CO and NCO levels increased to ~6.8% and ~5.5%, respectively in accordance with the increase in DSB levels in comparison with levels in WT cells (Fig. 3B). In *sml1Δ* mutants, the maximum levels of CO and NCO were slightly reduced in comparison with those in WT cells; however, the CO:NCO ratio exhibited a similar pattern as shown in WT cells. In contrast, NCO levels were similar in *mec1Δ* *sml1Δ* mutant and WT cells; however, the occurrence of COs was ~33% lower than that in the *sml1Δ* mutant (Fig. 3). Thus, Mec1^ATR^ is specifically required for CO formation, but not NCO formation (Fig. 3).

### Mec1^ATR^ and Tel1^ATM^ Cooperatively Promote the DSB-to-SEI Transition

To assess the progress of meiotic recombination in the absence of both Mec1^ATR^ and Tel1^ATM^, we constructed a homogeneous *pCLB2-TEL1* strain. The Tel1^ATM^ promoter was replaced by the *CLB2* promoter, a mitosis-specific allele activated during the mitotic cell cycle but suppressed in the meiotic cell cycle [40, 41]. In *pCLB2-TEL1* *mec1Δ* *sml1Δ* mutant cells, DSBs were formed at 2.5 h and their levels increased significantly in comparison with those in WT cells. Moreover, DSBs were not normally resected and were retained until 24 h (Fig. S2). Furthermore, SEIs and dHJs were not detectable upon 2D gel analysis in the *pCLB2-TEL1* *mec1Δ* *sml1Δ* mutant (Fig. S2). These results suggest that Mec1^ATR^ and Tel1^ATM^ are both required for the DSB-to-SEI transition during meiotic recombination.

### Mec1^ATR^ Promotes Recombination Independently of Rec8

**Fig. 4:**
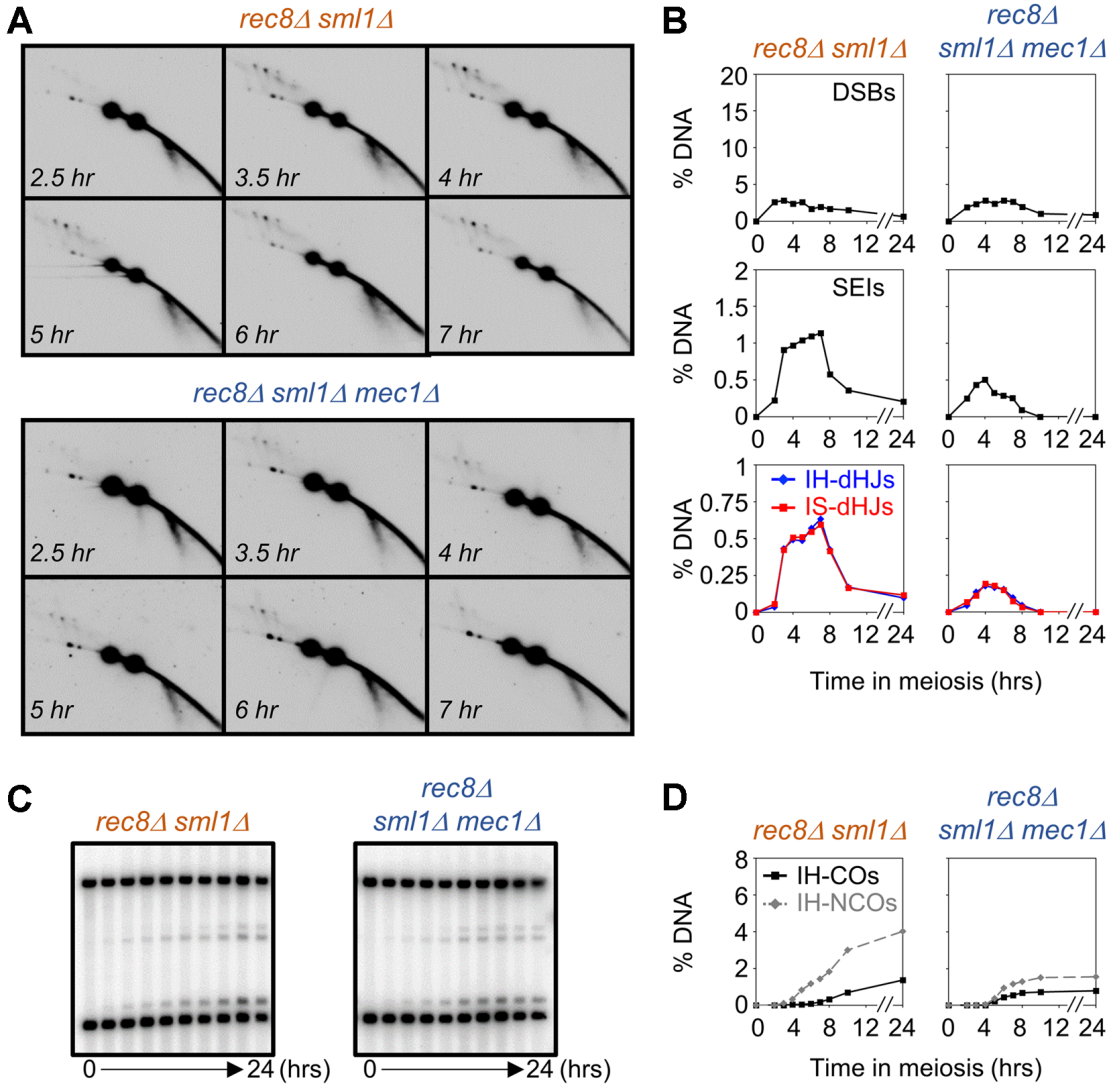
Cohesin-independent role of Mec1 in meiotic recombination. (**A**) Representative images of 2D gel analysis for *rec8Δ* *sml1Δ* and *rec8Δ* *sml1Δ mec1Δ*. (**B**) Quantitative analysis of DSBs, SEIs, IH-dHJs, and IS-dHJs in *rec8Δ* *sml1Δ* and *rec8Δ* *sml1Δ mec1Δ*. (**C**) Representative images of 1D and CO/NCO gel analysis for *rec8Δ* *sml1Δ* and *rec8Δ* *sml1Δ mec1Δ*. (**D**) Quantitative analysis of CO and NCO.

The meiosis-specific -kleisin subunit, Rec8, is required for sister chromatid axis formation, and the formation of appropriate synaptonemal complexes (SCs) [2, 42, 43]. Moreover, Rec8 is required for maintenance of homolog bias [2, 42]. In the absence of Rec8, the ratio of IH:IS-dHJ was ~1:1. Similarly, in the *mec1Δ* mutant, the interhomolog dHJs were reduced; however, the intersister dHJs increased in the 2D gel (Fig. 2). Furthermore, we assessed the association between Rec8 and Mec1 in *rec8Δ* *sml1Δ* and *rec8Δ* *sml1Δ mec1Δ* mutants (Fig. 4). At the *HIS4LEU2* hotspot, both *rec8Δ* *sml1Δ* and *rec8Δ* *sml1Δ mec1Δ* mutant displayed reduced DSB levels (Fig. 4B). Moreover, in the *rec8Δ* *sml1Δ mec1Δ* mutant, DSB turnover was delayed relative to that in the *sml1Δ mec1Δ* mutant (Fig. 4B). The IH:IS ratio in *rec8Δ* *sml1Δ* and *rec8Δ* *sml1Δ mec1Δ* mutants was 1:1 (IH:IS dHJs), consistent with the *rec8Δ* mutant phenotypes (Fig. 4B) [2]. Further, the *rec8Δ* *sml1Δ mec1Δ* mutant exhibited a reduction in the levels of CO and NCO (Figs. 4C and 4D). Thus, Mec1^ATR^ contributes to post-DSB stages independent of Rec8 during meiotic recombination.

Mec1^ATR^ and Tel1^ATM^ function in the DNA damage response, cell cycle checkpoint, and meiotic recombination. The present results elucidate the roles of Tel1^ATM^ in restriction of DSB formation and Mec1^ATR^ in CO-specific recombination at post-DSB stages during meiotic prophase. However, further studies are required to define the meiosis-specific effects of Mec1^ATR^ and Tel1^ATM^ during recombination and chromosome dynamics.

## Supplemental Materials



Supplementary data for this paper are available on-line only at http://jmb.or.kr.
