# Supplementary material for: Mec1 Modulates Interhomolog Crossover and Interplays with Tel1 at Post Double-Strand Break Stages
Source: J Microbiol Biotechnol. 2019 Dec 4;30(3):469–75. doi: 10.4014/jmb.1909.09020 (PMC9728206; doi:10.4014/jmb.1909.09020)
Supplement: Supplementary file 1 [file JMB-30-3-469-supple.pdf]

J. Microbiol. Biotechnol.
https://doi.org/10.4014/jmb.1909.09020

jmb

Supplementary information

Supplementary Figures

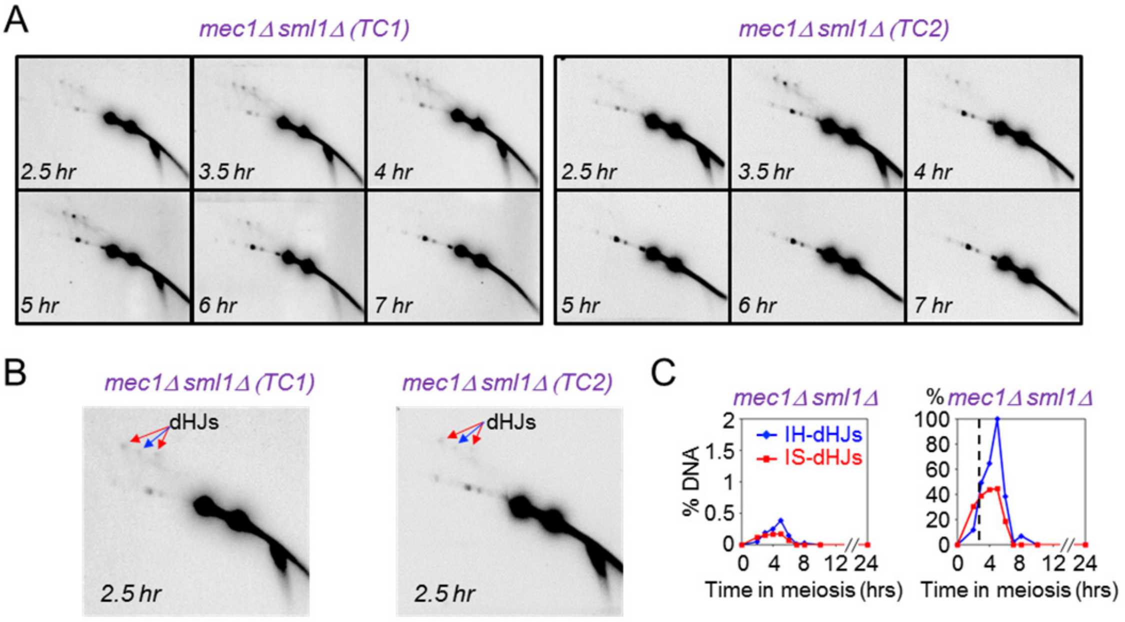

Fig. S1. Mec1 promotes interhomolog recombination. **(A)** Representative images of 2D gel analysis in an independent time course in the *mec1Δ sml1Δ*. **(B)** Representative images of 2D gel analysis at 2.5 h in the *mec1Δ sml1Δ*. Blue arrow, IH-dHJ; Red arrow, IS-dHJ. **(C)** Quantitative analysis of IH-dHJ and IS-dHJ in the *mec1Δ sml1Δ*.

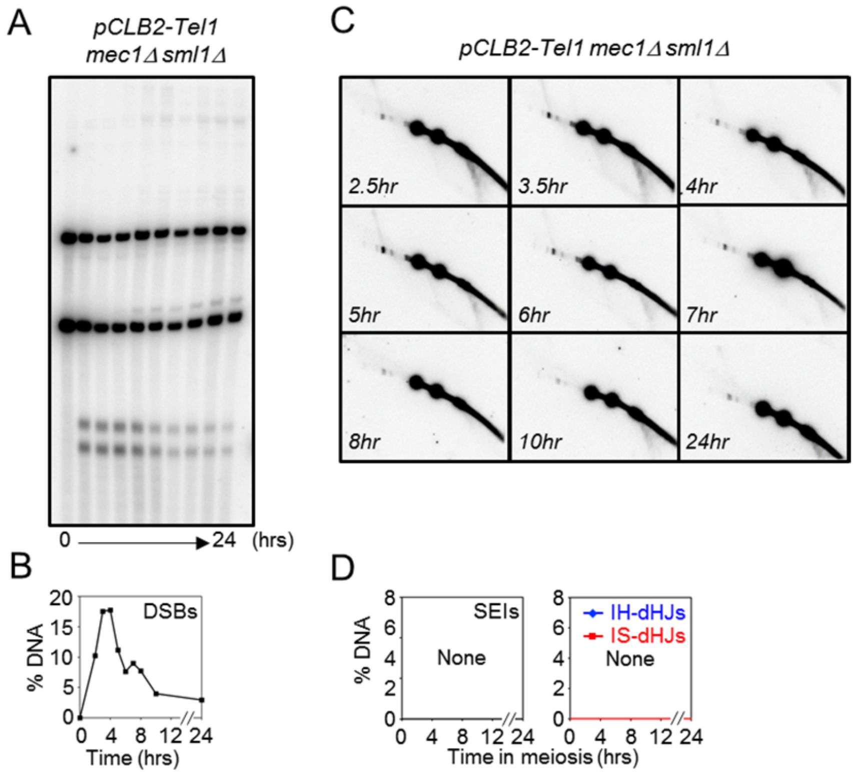

Fig. S2. DSBs and SEI formation in the *pCLB2-TEL1 mec1Δ sml1Δ* mutant. **(A)** Representative 1D gel image of the *pCLB2-TEL1 mec1Δ sml1Δ*. **(B)** Quantitative analysis of DSBs in the *pCLB2-TEL1 mec1Δ sml1Δ*. **(C)** 2D kinetic analysis of JMs using a 2D gel for the *pCLB2-TEL1 mec1Δ sml1Δ*. **(D)** Quantitative analysis of SEIs, IH-dHJs, and IS-dHJs in the *pCLB2-TEL1 mec1Δ sml1Δ*.

Supplementary Table

Table S1. Yeast strains used in this study.

Strain†	Genotype‡
KKY276	*MATa/MATα HIS4::LEU2-(BamHI+ori),his4-x::LEU2-(NgoMIV+ori)-URA3*
KKY275	*MATa/MATα HIS4::LEU2-(BamHI+ori),his4-x::LEU2-(NgoMIV+ori)-URA3, tel1Δ::KanMX/”*
KKY244	*MATa/MATα HIS4::LEU2-(BamHI+ori),his4-x::LEU2-(NgoMIV+ori)-URA3, sml1Δ::HygB/”*
KKY136	*MATa/MATα HIS4::LEU2-(BamHI+ori)/his4-x::LEU2-(NgoMIV+ori)-URA3, sml1Δ::HygB/”, mec1Δ::LEU2/”*
KKY414	*MATa/MATα HIS4::LEU2-(BamHI;+ori)/his4-x::LEU2-(NgoMIV+ori)-URA3, rec8Δ::KanMX/", sml1Δ::HygB/”*
KKY413	*MATa/MATα HIS4::LEU2-(BamHI+ori)/his4-x::LEU2-(NgoMIV;+ori)-URA3, rec8Δ::KanMX/", sml1Δ::HygB/”, mec1Δ::LEU2/”*
KKY416	*MATa/MATα HIS4::LEU2-(BamHI+ori)/his4-x::LEU2-(NgoMIV;+ori)-URA3, sml1Δ::HygB/”, mec1Δ::LEU2/”, pCLB2-TEL1::KanMX/”*
